# Prior pathogen exposure augments inter-individual heterogeneity in antibody levels and reinfection loads in a songbird-pathogen system

**DOI:** 10.1038/s41598-026-46682-9

**Published:** 2026-04-02

**Authors:** Jesse N. Garrett-Larsen, Anna A. Pérez-Umphrey, Arietta E. Fleming-Davies, James S. Adelman, Lauren M. Childs, Steven J. Geary, Kate E. Langwig, Dana M. Hawley

**Affiliations:** 1 Department of Biological Sciences, Virginia Tech, Blacksburg, VA USA; 2 Department of Biology, University of San Diego, San Diego, CA USA; 3 Department of Biological Sciences, University of Memphis, Memphis, TN USA; 4 Department of Mathematics and Virginia Tech Center for the Mathematics of Biosystems, Virginia Tech, Blacksburg, VA USA; 5 Department of Pathobiology & Veterinary Science, University of Connecticut, Storrs, CT USA

**Keywords:** Ecology, Ecology, Microbiology

## Abstract

Host responses to infection are often heterogeneous, with key consequences for pathogen transmission and evolution. Given the ubiquity of host re-exposure to the same pathogen, understanding how prior exposure alters inter-individual heterogeneity in disease responses is critical. Work in house finches (*Haemorhous mexicanus*) found that priming exposures to low or high doses of the bacterium *Mycoplasma gallisepticum* augmented host heterogeneity in susceptibility. Here we quantified whether priming similarly affects inter-individual heterogeneity in antibody responses, pathogen loads, and disease responses upon reinfection. Primed birds had more heterogeneous antibody responses prior to rechallenge relative to unprimed birds, with individual variation in antibody levels predictive of reinfection susceptibility. During rechallenge, pathogen loads, but not disease severity scores, were more heterogeneous in primed birds. However, when considering only birds successfully infected following re-challenge, priming did not significantly alter either mean pathogen loads or heterogeneity in loads. This suggests that priming effects on loads are largely explained by susceptibility differences. Nonetheless, because protective effects of priming limited sample sizes of successfully reinfected birds, further study is needed of whether priming influences load variability within successfully reinfected birds. Overall, prior exposure to pathogens alters inter-individual heterogeneity in several host traits, with implications for downstream infection dynamics.

**Supplementary Information:**

The online version contains supplementary material available at 10.1038/s41598-026-46682-9.

## Introduction

Individuals in a host population are rarely uniform in their responses to pathogen infection^1^. Instead, populations fall along a continuum from complete homogeneity—such that all individuals within a population respond similarly to the same pathogen—to strong heterogeneity—such that some hosts are completely resistant to infection while others are wholly susceptible (often termed all-or-nothing responses^2^). This inter-individual variation, also termed population-level heterogeneity, can arise from innate differences among individuals or from variable ecological conditions ^3^^4^. Regardless of the source, the heterogeneity of traits that mediate between-host dynamics, such as susceptibility (the probability of infection given exposure^2^), or infectiousness (the number of susceptible individuals infected by one infected host^5,6^) can have significant effects on epidemiological outcomes^5,7–9^ and host–pathogen evolution^10–12^. As a result, there is an increasing recognition of the utility of quantifying inter-individual heterogeneity in traits alongside traditional measures of central tendencies^13,14^. While substantial work has characterized population-level heterogeneity in singular processes (e.g., infectiousness, contact rates, susceptibility) and their epidemiological effects^3,5,6,8,15–18^, recent work shows that the epidemiological impacts of population-level heterogeneity in susceptibility versus transmissibility are distinct and dependent on potential correlations between the two^19^. This highlights the need to explicitly quantify how ecological factors concomitantly alter inter-individual heterogeneity in traits relevant to both susceptibility and infectiousness, which represent separate but related processes.

One important potential ecological source of inter-individual heterogeneity in host traits important for susceptibility and infectiousness is pathogen exposure history^15,20^. Individuals often vary significantly in infection-induced immune responses^21^, and the protection that prior exposure confers is often incomplete or wanes over time^22,23^. Recent work has shown that both vaccination^16^ and prior exposure to a pathogen^3^ can significantly increase the population-level variance in susceptibility, while simultaneously reducing a population’s overall mean susceptibility to reinfection. Further, the degree to which prior exposure augments heterogeneity in susceptibility may also depend upon the initial exposure dose^3^, particularly if low dose exposures produce less complete protection against reinfection than do higher priming doses^24,25^. Experimental studies often use high exposure doses to elicit replicable responses^24^, but, in nature, the exposure dose that hosts experience is more likely to vary widely, and often fall in the lower range^26,27^. However, it remains unclear whether exposure to different priming doses of pathogen induces underlying heterogeneity in immunological responses and subsequent within-host responses to infection. Understanding how pathogen exposure history, a relevant source of variation in many disease systems, influences the degree of inter-individual heterogeneity in host traits relevant to susceptibility and infectiousness is critical because of the documented consequences of such heterogeneity for outbreak risk^3,5,19^.

House finches (*Haemorhous mexicanus*) and their bacterial pathogen *Mycoplasma gallisepticum* (MG) are a well-characterized wildlife disease system that is particularly relevant for addressing these questions. In the early 1990s, MG jumped from poultry into eastern U.S. house finches, triggering an epizootic that caused substantial finch population declines^28^. MG infections cause mild to severe inflammation of the conjunctiva (conjunctivitis), and mortality is likely due to reduced anti-predator responses in infected birds^29^. Today, MG is endemic in almost all free-living house finch populations^30^, with seasonal outbreaks^31^. Pathogen load in the conjunctiva and disease severity are closely related in this system and serve as relevant proxies of transmission potential^30,32–34^. Transmission occurs primarily via the shedding of MG from the conjunctiva of infected birds onto fomites (e.g., bird feeders), which is then spread to conspecifics^35,36^. The viability of MG on fomites depends heavily on ambient conditions^37^. For these reasons, under natural conditions, house finches experience variable degrees and frequency of MG exposure. Further, while finches acquire significant protection from prior exposure to a range of pathogen doses^25,38^, immune protection in this system is incomplete^3,39^. As a result, reinfections are common^38,39^, and reinfected birds can transmit MG^25^. While work to date has used secondary challenge experiments to assess how prior MG exposure influences mean values of host responses such as pathogen loads, disease severity, and transmission potential, it is key to also understand how prior exposure specifically alters the degree of inter-individual heterogeneity in those traits. Indeed, recent work in this system demonstrated that prior exposure to MG augments population-level heterogeneity in susceptibility, and such heterogeneity in susceptibility can itself suppress pathogen outbreaks, above and beyond the protective effects of prior MG exposure on mean susceptibility^3,40^.

**Fig. 1:**
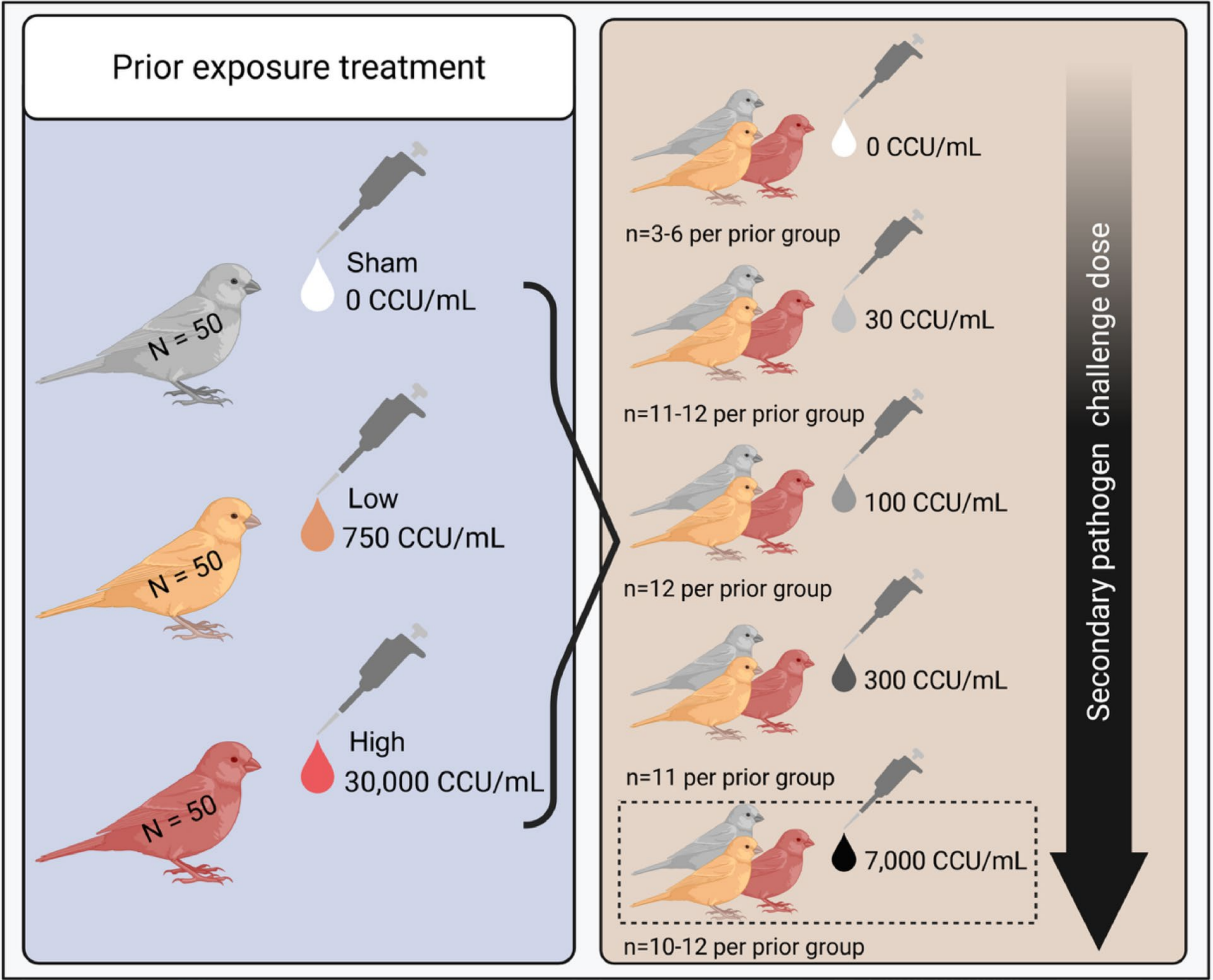
Experimental design to test how prior pathogen exposure (sham control, low dose, or high dose) influences host response heterogeneity upon secondary challenge. Birds were assigned to one of three priming exposure treatment groups, receiving either a sham (white), low (orange), or high (red) dose of *Mycoplasma gallisepticum*. Birds were allowed to recover from their initial infection and then were re-challenged with one of five secondary challenge doses. While antibody analyses were done for all secondary doses, all analyses of variation in transmission-relevant traits (disease severity and pathogen load) were limited to birds given a high-dose secondary challenge only (7000 CCU/mL; highlighted by dotted-line box) as this challenge dose produced the highest rates of successful reinfection, even within primed birds. Created in BioRender. Perez-Umphrey, A. (2026) https://BioRender.com/bxtgmef.

Here, we asked whether the extent of prior exposure to a pathogen alters inter-individual variation in several host traits relevant to susceptibility and infectiousness. We use data from a two-part experiment (Fig. 1), where animals with experimentally varied pathogen exposure histories (i.e., none, low dose, or high dose priming) were then challenged with one of five secondary doses. Data were collected for a marker of the adaptive immune response (antibodies) just prior to secondary challenge, as well as two transmission-relevant traits [disease severity (pathology) and infectiousness (pathogen loads)] in response to secondary challenge. These data were collected as part of a broader research effort to quantify heterogeneity in susceptibility, and so the overall experimental design and data collection process were the same as that reported in Hawley et al.^3^. However, in this study we analyzed distinct datasets from Hawley et al.^3^, whose work focused solely on the proportion of birds infected (0|1) in each treatment group (see *Methods*). Here, we analyze heterogeneity in metrics of disease severity (pathology) and infectiousness (pathogen loads) following secondary pathogen challenge to ask whether prior exposure augments heterogeneity in disease responses, both (1) in the challenged population as a whole, which captures the underlying distribution of susceptibility for the whole population based on pathogen load and (2) within successfully infected birds alone, which isolates heterogeneity of infectiousness in particular. We also asked whether heterogeneity in acquired immunity (IgY antibodies) was induced by initial pathogen exposure, and whether such heterogeneity in antibody levels just prior to challenge corresponded with patterns of population-level heterogeneity in susceptibility (previously documented in Hawley et al.^3^). We note here that, while the complexity of the immune system is not comprehensively represented by antibodies alone, they can be interpreted as a surrogate for broader immunological protection^41^.

To maximize the interpretability of our data, we picked a dual approach to quantifying inter-individual variation where we calculated both the Coefficient of Variation (CV) and a nonparametric alternative, Proportional Variability (PV)^42^ for all traits. PV is independent from the mean and bound between 0 and 1, allowing us to directly compare between traits on drastically different scales (e.g. pathogen load, eye score, and antibody levels). This enables us to robustly quantify variability while reducing biases that arise from small sample sizes and zero inflated data. Overall, we predicted that prior pathogen exposure would augment heterogeneity in each trait measured relative to hosts that were naïve at the time of challenge. We also predicted that inter-individual heterogeneity in antibody levels at the end of the primary challenge would be positively associated with previously documented population-level heterogeneity in susceptibility^3^ in response to secondary challenge. Finally, given prior work in this system^25^, we predicted that prior exposure to MG would induce higher pathogen load heterogeneity upon secondary challenge.

## Results

### MG exposure augments both mean values and antibody response heterogeneity

**Fig. 2:**
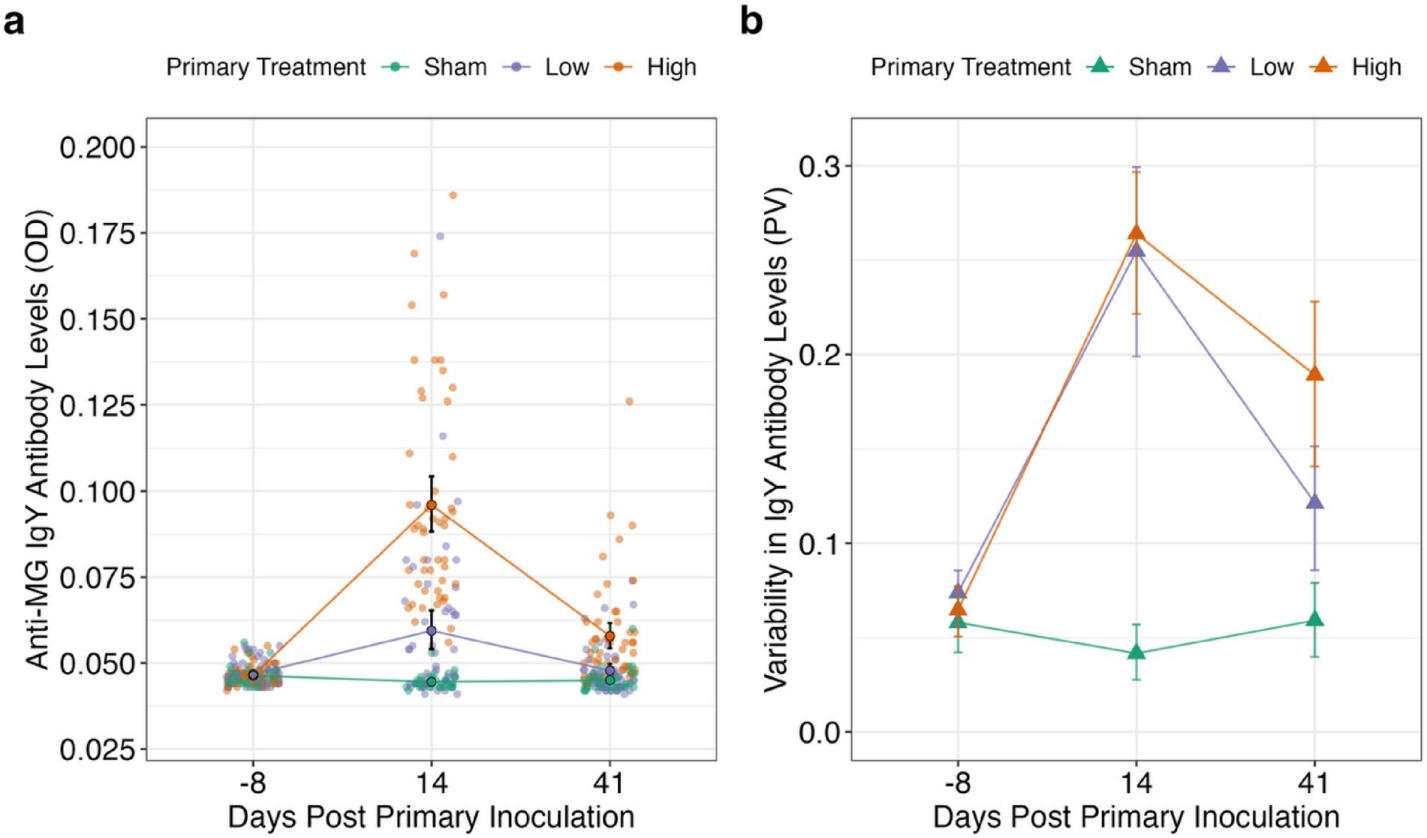
Antibody levels and their inter-individual variability in response to one of three priming exposure doses (sham control, low, or high) of *Mycoplasma gallisepticum* (MG). (**a**) Data points (translucent points) represent optical density (OD) antibody measures of individual birds, colored by primary treatment group (orange = “High”; purple = “Low”; green = “Sham”). Model predictions are represented by solid points and connecting lines and error bars represent ± 2SEM (SE of the model). X-axis represents days post-priming inoculation. Y-axis represents anti-MG antibody levels as measured by OD. ELISA OD readings have a lower bound of 0.04. **(b)** Triangles connected by lines represent the calculated proportional variability (PV) of each group with error bars representing 95% confidence intervals calculated by bootstrapping the data 1000 times. X-axis represents days post-priming inoculation. Y-axis represents variability in anti-MG antibody levels as measured by OD.

First, we asked whether initial exposure to a pathogen (at low or high priming doses) augments inter-individual variation in antibody responses. We found that exposure to MG induced antibody responses that were both higher on average, and more variable among individuals within the low and high priming groups. At both sampling points (here, 14- and 41-days post-priming inoculation [DPPI]), birds exposed to MG at either priming dose (low or high) had significantly elevated antibody levels relative to sham controls (Fig. 2, Supplementary Table 1). However, the impact of priming dose on inter-individual antibody variability changed over time. Antibody responses were equally variable between the low and high priming dose groups 14 days post-priming (Low priming PV = 0.255; n = 49; High priming PV = 0.264; n = 50), whereas birds in the high-priming group had more variable antibody levels by day 41 post-priming than those in the low-dose or sham-inoculated groups (Sham priming PV = 0.059, n = 50; Low priming PV = 0.121, n = 49; High priming PV = 0.189, n = 50; High vs. Low: F_1, 97_ = 6.29, p_adj_ = 0.014; High vs. Sham: F_1, 98_ = 17.4, p_adj_ = 0.0002; Sham vs. Low: F_1, 97_ = 7.34, p_adj_ = 0.012; Fig. 2). CV was also calculated to allow for comparisons with previous work^3^, and this metric indicated the same overall trends as PV in antibody data across the priming phase (Supplementary Fig. 1).

### Antibody levels predict reinfection probability

**Fig. 3:**
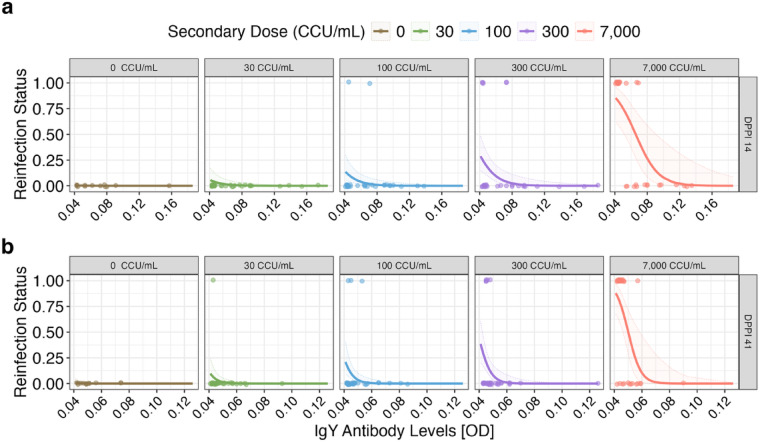
Individual variation in antibody levels on day 14 (**a**) or 41 (**b**) post-priming exposures to *Mycoplasma gallisepticum* (MG) predicts susceptibility to secondary challenge with one of five MG doses (facet labels), with reinfection status (y-axis) quantified as 0 (no) or 1 (yes). Model predictions (solid lines) ± 2SEM (SE of the model; shaded regions) of reinfection status (0|1; datapoints) for each secondary exposure dose on days post-priming inoculation (**a**) 14 and (**b**) 41. The x-axis represents antibody optical density [OD] values. Because secondary dose was an important predictor of reinfection likelihood, the five distinct secondary doses (facet labels) are shown separately.

Because antibody levels showed significant inter-individual variation in response to priming, we asked whether this variation was predictive of susceptibility to reinfection at the individual bird level. We used two independent generalized linear mixed effects models (Binomial distribution) across each post-priming sampling date (days 14 and 41 post-priming) to ask whether the antibody levels generated in response to the priming phase of the experiment were predictive of susceptibility upon secondary challenge. Because birds received distinct secondary challenge doses, log_10_ secondary dose was included in the model as a fixed effect. Antibody levels on both days post-priming 14 (E = − 71.21, df = 132, p = 0.003) and 41 (E = − 218.1, df = 146, p = 0.003), were predictive of susceptibility upon secondary challenge with MG, where birds with higher antibody levels had the lowest probability of infection upon secondary challenge (Supplementary Table 2; Fig. 3). As expected, the log_10_ secondary challenge dose was also predictive of susceptibility to reinfection, where birds challenged with a higher secondary dose were more likely to become reinfected in both models (DPPI 14: E = 1.95, df = 132, p < 0.001; DPPI 41: E = 1.79, df = 146, p < 0.001).

### Group-level variability in antibody levels parallels variability in susceptibility

**Fig. 4:**
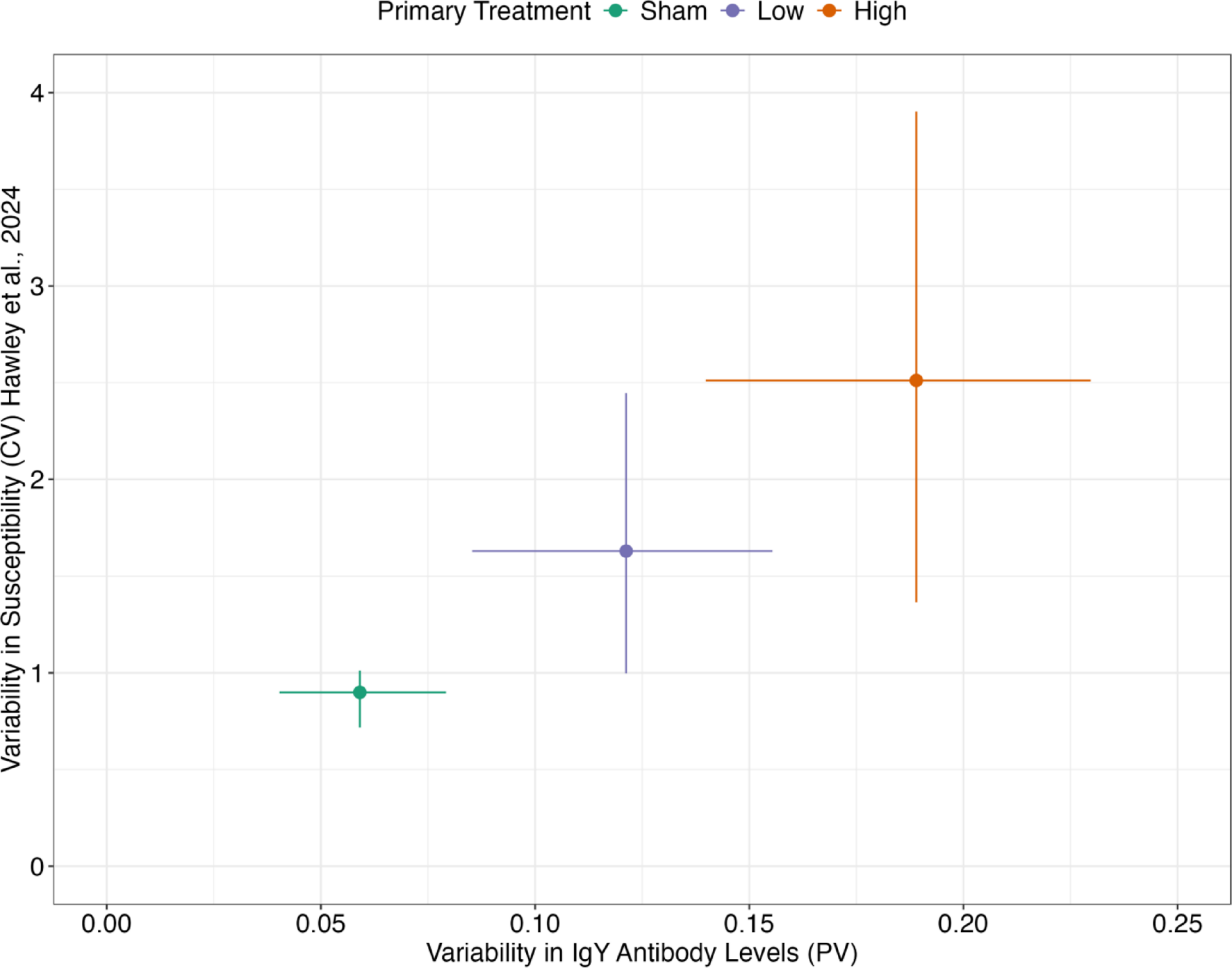
Group-level variability in antibody levels (x-axis) measured 41 days after inoculation with one of three priming pathogen doses (sham control, low, or high) qualitatively matches patterns in group-level heterogeneity in reinfection susceptibility (y-axis) upon secondary challenge on day 42 post-priming inoculation. Variability in reinfection susceptibility is shown as the gamma distributed coefficient of variation of susceptibility (CV; y-axis) reported in [15; https://doi.org/10.1371/journal.ppat.1012092]. Variability in antibody levels the day prior to secondary challenge (x-axis) is calculated as proportional variability (PV). Error bars represent 95% CIs calculated by bootstrapping the raw data 1,000 times with replacement. Shapes are colored according to the primary treatment group (green: sham; purple: low; orange: high).

Next, we asked whether the patterns in inter-individual heterogeneity in antibody levels that we detected (Fig. 2) matched those of heterogeneity in susceptibility quantified from the same experimental birds in previous work^3^. Indeed, the degree of variability (PV and CV) in antibody levels the day before reinfection challenge (DPPI 41) qualitatively matched the variability (CV) in susceptibility to reinfection following challenge on DPPI 42 (Fig. 4): birds that received a high priming treatment dose exhibited the greatest heterogeneity in antibody levels just before their secondary challenge (PV = 0.189; n = 50), and similarly showed the greatest heterogeneity in susceptibility when re-challenged (CV = 2.51 as reported in Hawley et al.^3^). Birds without any prior exposure (i.e., received a sham control dose during primary challenge) responded more homogeneously (in terms of both antibody levels and susceptibility) to the same series of secondary challenge doses that revealed heterogeneity in birds that had been primed with either low or high pathogen doses (Fig. 4).

### Prior exposure effects on mean and heterogeneity in pathogen loads

**Fig. 5:**
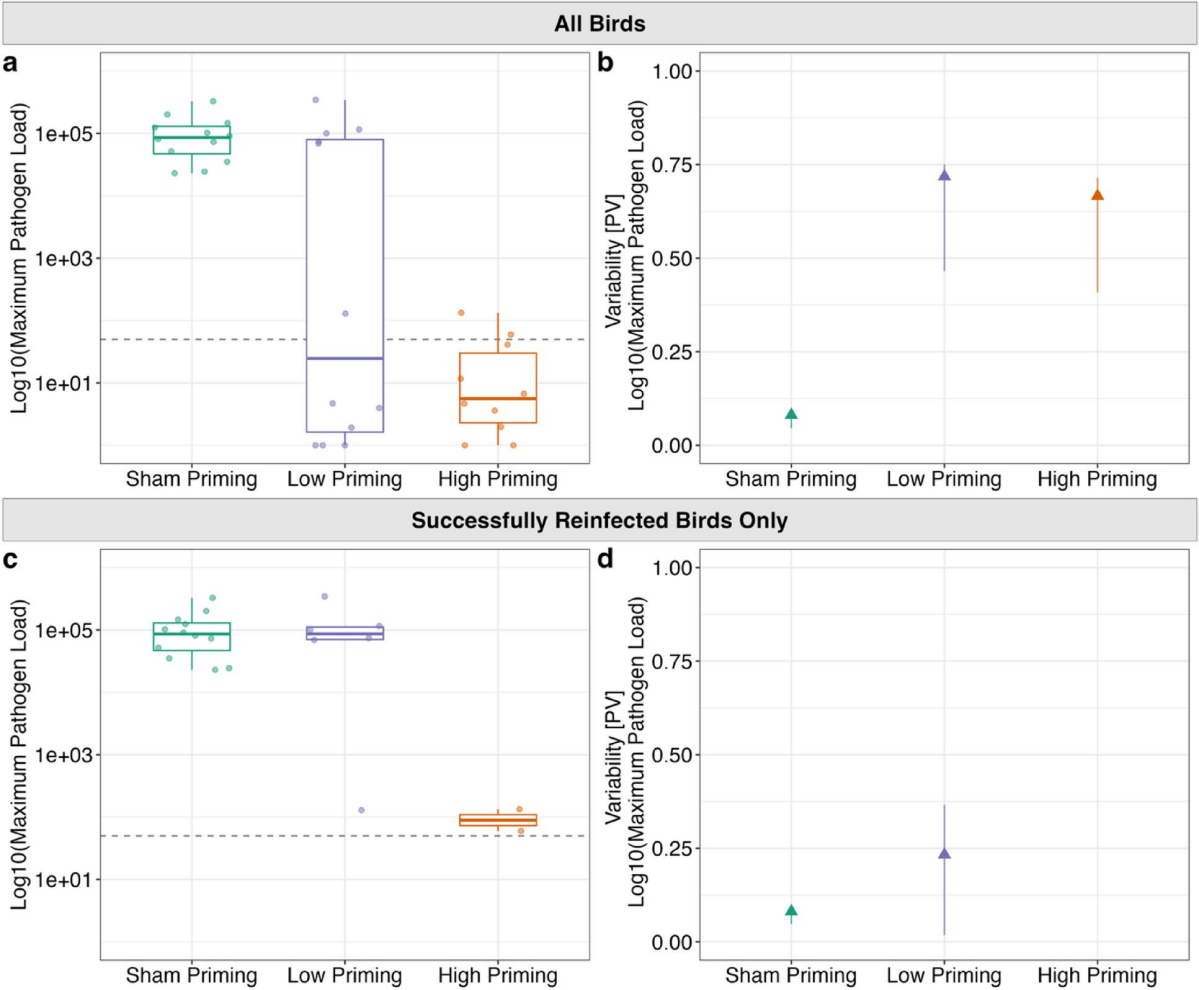
Maximum (**a**) pathogen loads and (**b**) their group-level variability for all birds from three priming treatments (x-axis) following high-dose secondary challenge, as well as maximum (**c**) pathogen loads and (**d**) their group-level variability for only successfully infected birds following challenge. Datapoints represent birds inoculated with the highest secondary dose of *Mycoplasma gallisepticum* (7000 Color Changing Units [CCU]/mL). Top two panels show the maximum (**a**) log10(pathogen load [*mgc2* copies + 1]) and (**b**) variability in maximum log10(pathogen load [*mcg2* copies + 1]) following secondary challenge for all birds rechallenged with MG. Bottom two panels show the maximum (**c**) log10(pathogen load [*mcg2* copies + 1]) and (**d**) variability in maximum log10(pathogen load [*mcg2* copies + 1]) following secondary challenge for only individuals that became infected when rechallenged with MG. Dashed gray lines in (**a**) and (**c**) represent the 50 copy cutoff used to determine successful infection. Note that no variability metrics are shown in (**d**) for the high priming group as only two individuals became infected. Box plot whiskers extend to values ± 1.5 × the IQR, center line represents the median, and solid, colored stars denote outliers. Colored triangles show proportional variability (PV) of disease responses calculated per treatment group. Error bars represent 95% confidence intervals for the PV calculated by bootstrapping the data 1,000 times with replacement.

We next asked whether priming induced inter-individual heterogeneity in pathogen loads (metric used was maximum pathogen load [*mgc2* copies] per individual, as determined through qPCR assay; see *Methods*) upon rechallenge. Here, we limited our analyses to only the highest secondary challenge dose (7000 CCU/mL), because other challenge doses resulted in low rates of reinfection (see Fig. 3), making it difficult to quantify heterogeneity in disease responses. First, we confirmed that prior pathogen exposure altered mean infection loads post-challenge in expected ways. As expected given our treatment design, prior exposure significantly predicted maximum log_10_ pathogen loads following challenge (X^2^ = 13.604, df = 2, p = 0.001; Fig. 5A). Post-hoc pairwise comparisons showed that, relative to unprimed sham controls, priming with either MG dose resulted in significantly lower maximum log_10_ pathogen loads following high-dose challenge (High vs. Sham Priming: Z = − 3.59, p_adj_ = 0.0005; Low vs. Sham Priming: Z = − 2.44, p_adj_ = 0.022; High vs. Low Priming: Z = − 1.26, p_adj_ = 0.31).

Prior exposure treatment also altered inter-individual heterogeneity in pathogen loads (Fig. 5B): birds with prior exposure to MG had more heterogeneous maximum log_10_ pathogen loads (Low priming PV = 0.72 [n = 12]; High priming PV = 0.67 [n = 10]) than birds with no prior exposure (Sham priming PV = 0.08 [n = 12]; Fig. 5, Supplementary Table 3). Brown-Forsythe tests revealed that each priming treatment resulted in a statistically distinct level of variability in maximum log_10_ pathogen loads, with the low-dose prior exposure group showing the highest variability (Low vs. High Priming: F_1, 20_ = 11.99, p_adj_ = 0.0037; Low vs. Sham Priming: F_1, 22_ = 22.16, p_adj_ = 0.00032; High vs. Sham Priming: F_1, 20_ = 4.95, p_adj_ = 0.038).

### Decomposing effects of prior exposure on susceptibility versus infectiousness

To isolate effects of prior exposure treatment on susceptibility versus the degree of host infectiousness once infected, we used a two-part hurdle analysis that separately estimates effects of prior exposure treatment on susceptibility to infection (0|1) and resulting pathogen loads within successfully infected birds only (susceptibility = 1). Compared to unprimed sham controls (Estimated probability of infection = 0.96, SE = 0.06), both low (Estimated probability of infection = 0.50, SE = 0.14, p = 0.045) and high (Estimated probability of infection = 0.23, SE = 0.13, p = 0.0082) dose priming exposures resulted in reduced susceptibility to infection. Within the subset of birds that became successfully infected following challenge (Sham: 12/12; Low: 6/12; High: 2/10), there were no significant differences in maximum log_10_ pathogen loads between prior exposure groups (X^2^ = 4.59, df = 2, p = 0.10; Fig. 5C).

To isolate how prior exposure influenced heterogeneity in infectiousness per se, we quantified heterogeneity in pathogen loads just within the subset of individuals that became successfully reinfected upon secondary challenge, again only considering birds in the highest secondary dose challenge group. Notably, we could not robustly estimate heterogeneity for the high-dose priming group, because only two birds became successfully reinfected after challenge. Reinfected birds with low-dose prior exposure to MG had a higher estimate of heterogeneity in maximum log_10_ pathogen loads (Low priming PV = 0.23 [n = 6]) relative to birds with no prior MG exposure (Sham priming PV = 0.08 [n = 12]), but this difference was not statistically significant (F_1, 16_ = 1.26, p = 0.28; Fig. 5D, Supplementary Table 3).

### Prior exposure effects on mean and heterogeneity in disease severity

**Fig. 6:**
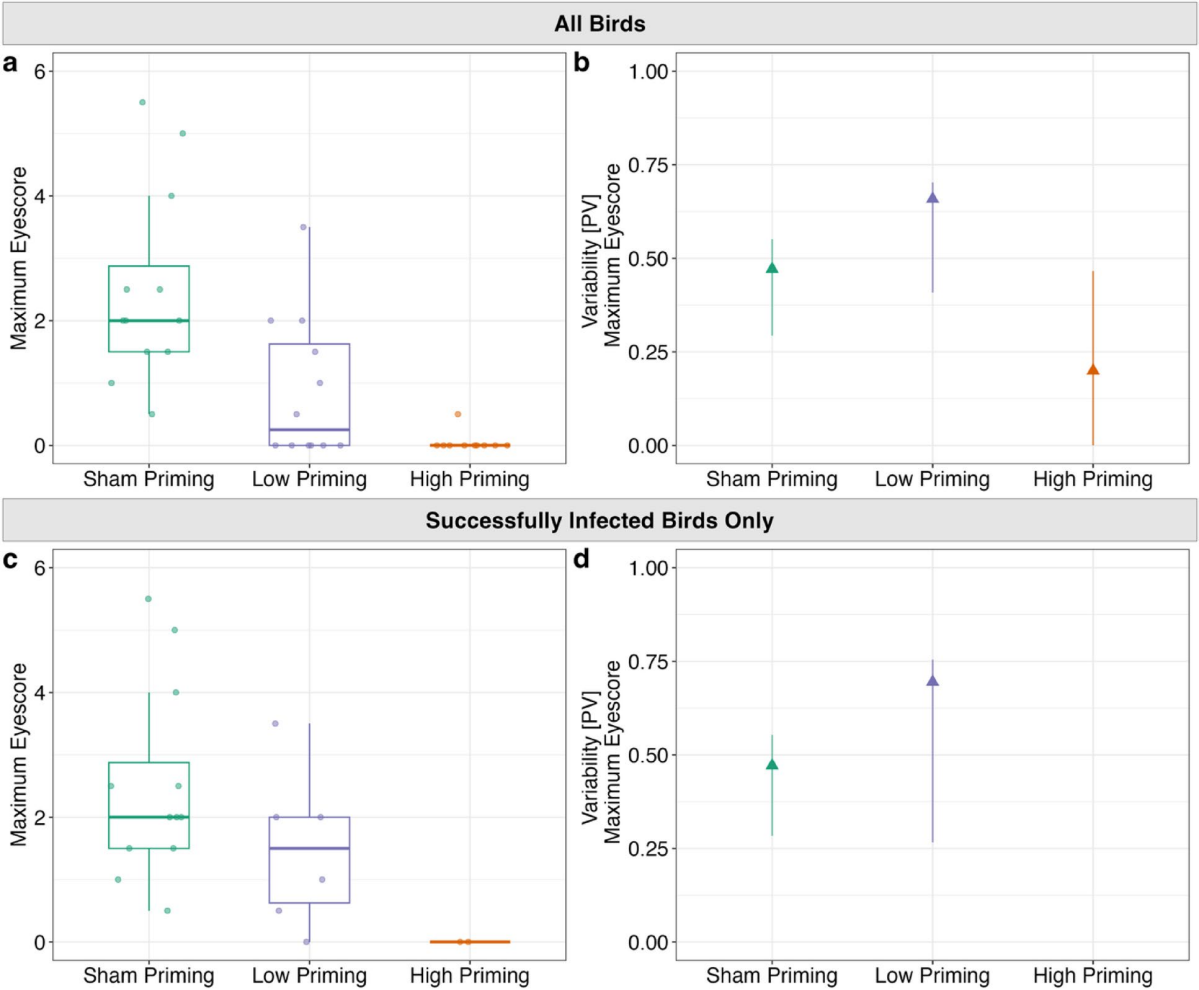
Maximum (**a**) disease severity scores and (**b**) their group-level variability for all birds from three priming treatments (x-axis) following high-dose secondary challenge, as well as maximum (**c**) disease severity scores and (**d**) their group-level variability for only successfully infected birds following challenge. Datapoints represent birds inoculated with the highest secondary dose of *Mycoplasma gallisepticum* (7000 Color Changing Units [CCU]/mL). Top two panels show the maximum (**a**) pathology and (**b**) variability in maximum pathology following secondary challenge for all birds rechallenged with MG. Bottom two panels show the maximum (**c**) pathology and (**d**) variability in maximum pathology following secondary challenge for only individuals that became infected when rechallenged with MG. Note that no variability metrics are shown in (**d**) for the high priming group as only two individuals became infected. Box plot whiskers extend to values ± 1.5 × the IQR, center line represents the median, and solid, colored stars denote outliers. Colored triangles show proportional variability (PV) of disease responses calculated per treatment group. Error bars represent 95% confidence intervals for the PV calculated by bootstrapping the data 1000 times with replacement.

We next considered effects of prior exposure treatment on mean disease severity (the metric used here was maximum eyescore per individual; see *Methods*) and heterogeneity in disease severity following high-dose challenge. Prior exposure significantly predicted maximum pathology (X^2^ = 19.06, df = 2, p < 0.0001), as expected. Post-hoc pairwise comparisons showed that either dose of prior exposure resulted in significantly lower maximum eye scores following challenge (High vs. Sham Priming: Z = − 4.30, p_adj_ < 0.0001; Low vs. Sham Priming: Z = − 2.73, p_adj_ = 0.01, High vs. Low Priming: Z = − 1.69, p_adj_ = 0.14), compared to control birds with sham prior exposure (Fig. 6A).

Heterogeneity in the maximum disease severity a bird exhibited post-secondary challenge was lowest in the high priming group (Sham Control PV = 0.47 [n = 12]; Low PV = 0.66 [n = 12]; High PV = 0.20 [n = 10]; Fig. 6B, Supplementary Table 3). Brown-Forsythe tests showed significant differences between the high prior exposure group and both the low and sham priming exposure groups in variability in maximum eyescore, confirming this result (Low vs. High Priming: F_1, 20_ = 7.22, p_adj_ = 0.021; High vs. Sham Priming: F_1, 20_ = 7.45, p_adj_ = 0.021). However, there was not a significant difference in variability between the low and sham control priming groups (F_1, 22_ = 0.22, p_adj_ = 0.64).

### Decomposing effects of prior exposure on susceptibility versus disease severity once infected

When analyzing only the subset of birds that were successfully infected following secondary challenge, we found that prior exposure still predicted maximum pathology (X^2^ = 6.26, df = 2, p = 0.044). Post-hoc pairwise comparisons showed that only birds with high dose prior exposure had significantly lower pathology than those with sham control prior exposure (High vs Sham Priming: z = − 2.39, p_adj_ = 0.026; Fig. 6C). The maximum disease severity post-challenge showed no differences in variability between sham and low-dose priming groups (F_1, 16_ = 0.026, p = 0.88; Fig. 6D, Supplementary Table 3) when considering successfully infected birds only.

## Discussion

Quantifying inter-individual heterogeneity in traits relevant to host–pathogen interactions can present novel insights beyond those gained from mean values alone^43^. In particular, heterogeneity in host traits relevant to pathogen fitness can have key impacts on downstream epidemiological^5^ and evolutionary^44^ dynamics. Because host populations are typically composed of individuals with variable infection histories and acquired protection^21^, it is important to understand how prior pathogen exposure modulates the degree of inter-individual heterogeneity in host traits that underlie immunological protection and pathogen spread^20^. Here, we used data from a secondary challenge experiment to determine whether pathogen exposure history induces heterogeneity in host disease traits relevant to susceptibility and infectiousness. Additionally, we were able to compare heterogeneity in a readily measurable immune marker of prior exposure to estimates of heterogeneity in susceptibility presented in previous work^3^ but generated from the same birds used here. We show that, relative to a pathogen-naïve population (sham controls), hosts with prior exposure show significantly higher inter-individual heterogeneity in an immunological trait relevant to susceptibility, with patterns of heterogeneity in antibody levels qualitatively matching previously quantified values of population-level heterogeneity in susceptibility. Prior exposure similarly augmented heterogeneity in pathogen loads for challenged birds as a whole, but such heterogeneity appears to be driven largely by differences in susceptibility to challenge versus variation in infectiousness.

Our primary objective was to quantify whether prior exposure to pathogens alters the degree of inter-individual variation in host responses. We first examined a marker of immunological protection and found that initial exposure dose was positively associated with group-level variability in IgY anti-MG antibody levels directly before secondary challenge. In turn, this individual variation in antibody levels was predictive of reinfection probability, consistent with previous work in this system^38^. This suggests that prior exposure to pathogens does not just alter the average level of protection in a population, but can also modulate the degree of variation in acquired protection that is present, which can have key population-level impacts for disease spread^8,14,16^. Interestingly, we found that the extent to which prior exposure augmented variability in antibody responses at the later sampling timepoint (DPPI 41) depended on the dose of prior exposure, while the degree of variability in antibody responses was similar between prior exposure doses for the earlier timepoint (DPPI 14) representative of peak responses. This change in patterns of heterogeneity across priming doses appears to arise because birds inoculated with a high priming dose maintained higher mean levels of antibodies across post-priming sampling periods than those inoculated with a low dose. This suggests that the kinetics of antibody responses post-exposure may be important to account for when determining to what degree prior exposure to pathogens augments inter-individual variability in antibody responses. House finches acquire incomplete but significant protection from prior exposure to MG, and birds that are exposed to a higher priming dose generate higher mean antibody levels, as we show here and in prior work^24,38^. But antibody levels wane over time in this system^39,45,46^. This combination of incomplete initial immunity, even from high priming doses^39^, and waning immunity over short time scales may be a mechanism by which prior exposure generates heterogeneity in antibody and subsequent disease responses.

The ability to link easily measurable biomarkers to population-level variation in traits relevant to susceptibility can help to broaden the use of models that incorporate heterogeneity in susceptibility to inform management decisions^47^. Pathogen-specific antibodies are widely applicable, relevant, and convenient biomarkers that have been used for decades as proxies for estimating standing immune protection^48,49^. Here, we add to the growing evidence that the magnitude and durability of antibody responses is positively associated with prior exposure dose in this system and others^38,50^, and that antibody levels just prior to inoculation are predictive of protection from reinfection^38,39^. Importantly, the primary dose that an individual received was intentionally not included in our models that compared antibody levels with reinfection probability (Supplementary Table 2). Therefore, without knowing the dose of pathogen that individuals were exposed to, we could still predict group level susceptibility to secondary challenge based solely on antibody levels that were collected after birds had resolved their infection.

Inter-individual heterogeneity in antibody levels has been studied in the context of the recent SARS-CoV-2 pandemic, showing significant variability in antibody titers between groups (age, sex, vaccination status)^48^. In another study, within-group variability in antibody titers was associated with functional differences in immunity distinct from absolute antibody concentration. Specifically, individuals with maximum antibody titers above the group median exhibited qualitatively different functions (e.g. higher neutralizing antibody titers, antibody-dependent complement deposition, and antibody-dependent neutrophil phagocytosis) compared to those below the median^51^. Thus, it is important to not only measure central tendencies, but also variability, as focusing solely on central tendencies can obscure biologically important variation in immune function. Indeed, variability in antibody levels may be associated with variation in other aspects of acquired immunity through their effector functions (opsonization, Fc-mediated phagocytosis, antibody-dependent complement activation^51^), even if not explicitly predictive of cellular responses^52^.

While we did not directly measure antibody effector functions here, we were able to compare inter-individual variation in antibody levels with that of previously measured functional variation in susceptibility, defined here as the likelihood of infection given a known dose of pathogen. Population-level heterogeneity in susceptibility is measured through resource intensive dose–response experiments^3,16,43,53^. These experiments are valuable in that they allow for an assessment of variability in susceptibility that is independent of the population’s mean^43^, and epidemiological models that account for such heterogeneity in susceptibility more accurately predict outbreak size^8^. However, these dose–response experiments and subsequent quantification of heterogeneity in susceptibility are not typically feasible with field-collected data. We found that prior exposure dose produced parallel effects on heterogeneity in antibody levels and previously measured heterogeneity in susceptibility in this system^3^. While this relationship is correlative and generated from only three priming dose groups, it raises the possibility that measures of antibody heterogeneity from field-sampled birds could potentially be used as a coarse proxy for heterogeneity in susceptibility in place of intensive dose–response experiments. Future research with more treatment groups is needed to determine whether antibody response heterogeneity is in fact a robust proxy for population-level heterogeneity in susceptibility in this system, and potentially others. Research could also compare the utility of field-collected antibody responses versus dose–response experimental data for parameterizing susceptibility distributions for Susceptible-Infected-Recovered (SIR) epidemic models e.g.^3,16^.

The second main goal of our study was to understand whether prior exposure to MG augmented heterogeneity in traits relevant to transmission, such as pathogen loads and disease severity. We first quantified heterogeneity in pathogen loads and disease severity for all birds challenged with the highest secondary dose, regardless of whether or not a given individual was detected as successfully infected following secondary challenge. This analysis allowed us to consider the full range of relevant trait variation present in a population given a known high-dose exposure. It also allowed us to account for potentially transient, low-level infections that were not detected by our periodic sampling and our conservative infection threshold (> 50 copies; see *Methods*) but still represent biologically meaningful inter-individual variation in infection responses. The analysis of all challenged birds found that both low and high dose prior exposure led to higher inter-individual variation in log_10_ maximum pathogen load upon rechallenge, relative to birds with no prior exposure. Because pathogen loads during reinfection were recently shown to predict the likelihood of spread to a naive cagemate^25^, the increased variation in loads among primed individuals that we detected here should act to further reduce subsequent outbreak size in primed populations, along with reductions in mean susceptibility and increases in variation in susceptibility^3^.

Interestingly, effects of prior exposure on heterogeneity in disease severity in our study showed a different pattern, with a significant reduction in inter-individual variability in disease severity in the high prior exposure group. This reduction appeared to be driven by almost complete protection from reinfection in the high priming group, consistent with the stronger acquired protection against disease versus pathogen loads documented previously in this system^25,45^. House finches in Virginia, the source population for the birds used in our experiment, have evolved tolerance to infection^30^ (minimizing pathology per pathogen load). This tolerance may have constrained the range and maximum disease severity expressed within our host population, potentially limiting our ability to detect effects of prior exposure on inter-individual variability in disease severity. If this were the case, in systems or populations with less evolved tolerance, there may be more variability in responses to rechallenge. On the other hand, because prior exposure or vaccination can produce stronger protection against disease versus infection load in many systems e.g.^54^, it is possible that acquired protection generated from prior exposure, particularly to high priming doses, generally reduces inter-individual variation in disease severity by producing more complete disease protection. The distinct effects of priming on within-group heterogeneity for pathogen loads versus disease severity, which are both predictive of transmission success in this system^25,32^, highlights the importance of considering the underlying mechanisms by which acquired protection from prior exposure acts on diverse host traits, akin to infection versus disease-blocking vaccines^55^.

It is also key to disentangle whether effects of priming on group-level heterogeneity in downstream responses stem from variable effects of prior exposure on susceptibility to infection, the degree of infectiousness or disease once infected, or both^19^. We addressed the distinction between these two processes by using a hurdle analysis to first determine how prior exposure treatment altered infection probability. As expected, based on prior work^3,38^, prior exposure treatment significantly predicted reinfection probability, with the high priming group showing the lowest mean susceptibility (23% chance of infection versus 96% chance for individuals with no prior exposure). As a result of this reduced susceptibility, we had low sample sizes of successfully infected birds in our priming groups for determining how prior exposure altered mean host responses and group-level heterogeneity. However, even within our small group of successfully infected birds, we detected significant effects of prior exposure treatment on maximum disease severity. However, there was no support for reductions in mean pathogen load due to prior exposure treatment, once differences in reinfection likelihood were accounted for by our hurdle model. These results are consistent with “all-or-nothing” load responses documented in other host–pathogen systems^2,16^, whereby hosts acquire either strong protection from prior exposure or virtually no protection, leading to mean pathogen loads in low-dose primed birds that became infected that were equivalent to those of unprimed hosts. Intriguingly, a study in the same host–pathogen system using a distinct type of priming (repeated, low dose exposures) showed qualitatively similar patterns, with pathogen loads of primed birds showing a bimodal distribution following challenge (Fig. 2B, Leon et al.^25^).

To isolate effects of prior exposure on heterogeneity in infectiousness in particular, we also quantified heterogeneity in pathogen loads and eye scores within the subset of birds that became successfully infected following challenge. Given the protective effects of prior exposure on reinfection likelihood, we only had sample sizes of successfully infected birds that were sufficiently high for estimating heterogeneity in our low-dose primed and control groups. Here, in contrast to the results for all challenged birds, we found no statistical support for higher heterogeneity in pathogen loads in the low-dose primed group relative to the control group, though estimates of heterogeneity were slightly higher in the low-dose priming group. This suggests that the strong detected effects of prior exposure on pathogen load heterogeneity for all challenged birds, regardless of whether or not they became successfully infected, is largely a function of variation in susceptibility^3^ versus heterogeneity in infectiousness once infected. However, given the limited sample sizes of successfully infected birds even in our low-dose priming group (n = 6), further study is needed to determine whether priming has effects on heterogeneity in loads beyond those associated with initial susceptibility. Overall, decomposing these two biological processes highlights that heterogeneity in susceptibility and infectiousness should be estimated separately, as they can represent independent biological processes with potentially distinct or combinatorial epidemiological consequences^19^.

In conclusion, we found that prior exposure to MG augments variability in both antibody responses during priming and in infection loads for all challenged birds. Notably, the detected variability in infection loads induced by priming appears to be driven by susceptibility mechanisms, where prior exposure appears to have an all-or-nothing effect on pathogen loads in which individuals with low prior exposure either resisted infection or developed pathogen loads similar to birds with no prior exposure. While past studies considered heterogeneity in transmission more broadly^5^ and in pathogen loads in particular^56–58^, future work should investigate specifically how heterogeneity in traits such as pathogen loads affect pathogen spread, as well as further decompose to what extent such heterogeneity is generated by variation in susceptibility to infection and/or infectiousness. Practically, because we found that inter-individual heterogeneity in antibody responses matched patterns for population-level heterogeneity in susceptibility, our results also suggest that quantification of pathogen-specific antibodies may be a potential way to estimate population-level heterogeneity in susceptibility in some systems. While this is an intriguing possibility, future work should focus on validation of this approach with larger numbers of experimental groups. Ultimately, with validation, such approaches could allow easier incorporation of variation in infection-induced immunity into mathematical models^21^, as researchers are increasingly doing^59,60^. Given that many host–pathogen systems, including those of humans, are characterized by incomplete acquired protection^61–63^, understanding how prior exposure alters the degree of variation among hosts is critical for predicting both outbreak dynamics and the selection pressures acting on pathogens in populations of hosts with variable exposure histories.

## Methods

### Ethics statement

All bird handling, husbandry, and experiments were approved by the Virginia Tech IACUC (protocol #21–082). Capture and collection of house finches was approved by the Virginia Department of Game and Inland Fisheries (066,646) and USFWS (MB158404). Authors complied with all relevant guidelines and regulations including the ARRIVE guidelines.

### Data overlap statement

The data analyzed in this paper were collected as part of the same experiment published in Hawley et al.^3^. All data were collected from the same birds, and the inclusion criteria for birds and qPCR positivity threshold for determining successful infection (see below) are identical between the two studies. The current work, however, analyzes completely distinct datasets (antibody levels, quantitative variation in load, and variation in disease severity) from that analyzed in Hawley et al.^3^, which only analyzed group-level proportions of infected birds across treatment groups. The only data that are used in common between the two papers include: (1) infection success (0 | 1) in response to challenge was analyzed here at the individual level versus at the group level in Hawley et al.^3^; (2) eye scores were analyzed here to quantify heterogeneity among individuals, and the same scores were used in Hawley et al.^3^ to parameterize mortality rates for our epidemiological model; (3) estimates of heterogeneity in susceptibility (as CVs) with error bars generated from bootstrapped values in Hawley et al.^3^ are presented visually here in Fig. 4 as a qualitative comparison to estimates of variability in antibody levels. However, no statistical comparisons were performed on the estimates of heterogeneity in susceptibility in this paper. Finally, there are no identical figures or text between the two papers, though both papers include distinct figures of the experimental design, each adapted to illustrate the experimental design relevant to the analyses performed in their respective studies.

## Experimental design

To ask whether prior pathogen exposure history determines the degree of variation in host traits relevant to transmission dynamics (antibody responses, pathology, and pathogen loads), we designed a fully factorial 3 × 5 two-phase experiment, where we first experimentally varied host prior pathogen exposure, allowed for complete infection recovery (forty-two days), and then re-exposed birds using a gradient of secondary challenge doses. Free-living, hatch-year house finches were captured from the wild in Montgomery County, VA, USA, and confirmed to be pathogen-naive (n = 150; see Supplementary Methods for capture and quarantine details) prior to assignment to one of three primary treatments (exposure doses: sham [n = 50], low [n = 50], or high [n = 50]). For secondary challenge, birds from each primary treatment group were assigned one of five secondary exposure doses (Fig. 1). Birds were stratified by sex and randomly assigned to priming and secondary treatment groups within sex to ensure equal sex ratios between groups.

## Sample & data collection

### MG IgY antibodies

We collected blood samples (~ 80 µL) from each bird by brachial vein puncture with a sterile 26-gauge needle. We collected blood using heparinized capillary tubes which was then expelled into 1.5 mL centrifuge tubes and kept on ice until centrifugation (13,000 × RPM for 7 min) to separate plasma from red blood cells. Plasma samples were stored at − 20 °C until use in ELISA assays. We used a commercially available ELISA kit (FlockChek *M. gallisepticum* antibody enzyme-linked immunosorbent assay kit [IDEXX, Wesbrook, Maine, USA]) to measure house finch anti-MG IgY antibodies from serum samples. We followed the protocol outlined in Hawley et al.^64^, using a 0.061 OD (optical density) seropositivity threshold cutoff. Sixteen plasma samples were lost during processing (see Supplemental Methods). Importantly, fourteen of these were from sham inoculated birds which would have been seronegative, having never been exposed to MG. Had these fourteen individuals been included in our logistic regressions, our effect sizes would be even more pronounced.

### Pathology

We assessed clinical status according to eye lesion severity, which was scored on a 3-point scale in 0.5 increments (0 = no detectable swelling or inflammation; 1 = minor swelling around the ring of the eye; 2 = moderate swelling and eversion of the conjunctival tissue; 3 = eye nearly hidden by swelling and crusted exudate^46^), and summed across both eyes for a composite score. The same experienced observer collected these data while blind to a given bird’s treatment group to eliminate observer-bias.

### Pathogen load

Following eye scoring, we swabbed the conjunctiva of each eye with a sterile cotton tipped applicator and then vigorously swirled in tryptose phosphate broth (TPB) for 5 s. The left and right eyes of each bird were swabbed separately and then pooled into a tube containing 0.3 mL TPB. Swabs were discarded and the sample solution was kept on ice and stored at -20 °C until DNA extraction. We extracted MG DNA from the TPB solution using 96-well Qiagen DNeasy Blood and Tissue Kits (Qiagen, Hilden, Germany). Pathogen load was quantified via quantitative real-time PCR (qPCR; QuantiNova Kit [Qiagen, Hilden, Germany]) on a QuantStudio 5 using probes, primers, and plasmid standards with *mgc2* gene insert^64,65^. All samples were run in triplicate alongside a standard curve following Hawley et al.^66^.

Pathogen loads were analyzed as log_10_(*mgc2* copies + 1); to account for zeros in the dataset. This is referred to simply as log_10_ pathogen load in this text. For analyses relating antibody levels or prior exposure treatment to reinfection susceptibility, we conservatively defined infection as > 50 MG pathogen copies at any time point post-secondary challenge^3^. Because birds may be asymptomatically infected^67^, pathology alone was not used to define infection status (sample sizes: Supplementary Methods; Supplementary Table 4).

### Data analysis

All data were analyzed in RStudio (v4. 3. 1; R Core Team 2023). Model selection was performed using Akaike’s Information Criterion adjusted for small sample sizes (AICc^68^).

### Measures of heterogeneity

We estimated inter-individual heterogeneity of all measures of interest (antibody levels, pathogen load, and disease severity) using proportional variability (PV), a measure of variation within groups that is independent of the mean^42,69^. In contrast to Pearson’s coefficient of variation (CV), PV is less sensitive to rare events, does not assume a normal distribution, and, importantly for our dataset, is not biased by zero-inflation and nonparametric data^70^. Initially, CV was preferred as it accounts for the tendency of variance to increase with the mean. Recent work has suggested that the CV introduces bias when rare events are included or the mean approaches zero, therefore, several new approaches to calculate dispersion independently of measures of central tendencies have been developed^42,69,71–73^. The PV is useful for comparisons of variability between measurements on different scales as it is a true proportion bound between 0 and 1 and it accounts for strongly disparate patterns exhibited by different groups^74^. This metric is particularly suited to quantify the degree of inter-individual variation in continuous host traits, such as antibody levels or pathogen loads. In contrast, PV is not suited to measure variability in binomial data such as infection status or susceptibility, as in these cases variability is inherently tied to the mean. We calculated PV using the R package *CValternatives*^75^. We also calculated CV for some analyses in order to draw comparisons with previous analyses^3^, and contrast with PV to robustly quantify variability.

To compare measures of variability between groups and over time, we estimated 95% confidence intervals by manually bootstrapping the raw data 1000 times per group with replacement. We used the 2.5th and 97.5th percentiles of the distribution of the bootstrapped data to estimate the 95% confidence intervals for each variability metric. Additionally, Brown-Forsythe tests, which compare the amount of absolute deviation from the median within each group^76^, were used to assess differences in variability across treatment groups. Importantly, the Brown-Forsythe test was developed as an alternative to Levene’s test to be robust to non-normal distributions^77^. Post-hoc analyses were then performed using pairwise Brown-Forsythe tests with Benjamini–Hochberg corrections for multiple comparisons.

### Variability in host responses: antibodies

We first asked whether prior exposure induced differences in the mean or heterogeneity in antibody levels in a degree-dependent manner (none, low, or high prior exposure), and whether this heterogeneity matched documented patterns of heterogeneity in susceptibility during a secondary challenge^3^. Variability metrics (PV and CV) were calculated for antibody levels within each primary treatment group per day (DPPIs -8, 14, and 41) over the primary phase of the experiment. We then asked whether individual variability in antibody levels during the priming phase (measured at two distinct time points) were predictive of reinfection susceptibility. We used a generalized linear mixed effects model (GLMM; *glmmTMB*^78^) with a Gamma distribution and log link to test whether the interacting fixed effects of primary inoculation dose (categorical: none, low, or high) or days since primary inoculation (DPPI; factor with levels − 8, 14, and 41) predicted IgY anti-MG antibody levels (reported as ELISA optical density [OD] values). Individual bird identity was included as a random effect to account for repeated measures. Additionally, dispersion was modeled as a function of DPPI and primary treatment. Model selection was performed using Akaike’s Information Criterion (AICc; aictab, *bblme*). The model was fit using maximum likelihood estimation, with significance assessed using Wald z-tests with a significance threshold of ɑ = 0.05. Nonsignificant fixed effects were removed from the model. After fitting the model, we conducted post-hoc pairwise comparisons to explore the effects of different primary inoculation doses on each day post inoculation. Comparisons were performed using Tukey’s adjustment for multiple comparisons (emmeans^79^), to determine differences in estimated marginal means at each day post inoculation.

To ask whether there was a relationship between IgY antibody levels during the priming phase and susceptibility to reinfection in the secondary phase, we fit two independent GLMMs with binomial distributions and a logit link for DPPIs 14 and 41. We asked whether antibody levels, secondary dose (transformed as log_10_ + 1; referred to as log_10_ secondary dose), or their interaction were predictive of reinfection susceptibility. No significant interaction was evident between antibody levels and log_10_ secondary dose and therefore the interaction term was removed from the models. Likewise, bird sex was not predictive of antibody levels or susceptibility upon reinfection and was therefore excluded from the final models. Models were fit using maximum likelihood estimation. Significance was assessed using Wald z-tests with a significance threshold of ɑ = 0.05. Model selection was performed using Akaike’s information criterion (AICc) values.

### Variability in host responses: pathogen load & pathology

The second part of our analyses focused on whether prior exposure induces inter-individual variability in traits relevant to infectiousness (eye score and pathogen load). As part of the second phase of our experiment, birds were re-challenged with one of five secondary doses. Due to the protective effects of prior exposure, there were few successful reinfections in the four lowest secondary doses, and so we limited our analyses to those birds that received a high secondary dose (7000 CCU/mL) of which there were successful reinfections (n = 20/34). While not all individuals re-challenged with the 7000 dose became reinfected, we included all individuals in our initial analyses, regardless of reinfection status to maintain all biologically relevant variability and unobserved heterogeneities^80^. In the wild, all individuals have the possibility of re-exposure, with resistance to reinfection representing an important outcome for outbreak dynamics (e.g. all-or-nothing immunity^2^). Nevertheless, including resistant birds in our analyses of heterogeneity in host responses may artificially inflate heterogeneity estimates. Therefore, we present two complementary analyses. First, we analyze variability in all individuals regardless of reinfection status to maintain all unobserved heterogeneities. PV was calculated using each bird’s maximum eyescore and maximum log_10_ pathogen load during secondary challenge (DPSI 4, 7, 14, and 21). We also tested for mean differences in maximum pathogen load and eyescore using a Kruskal–Wallis test^81^, followed by a pairwise Dunn’s test with Bonferroni adjustments when Kruskal–Wallis showed significance^82^.

Next, we used a two-part hurdle analysis to ask whether priming treatment affected probability of reinfection (1|0), and whether, of the successfully reinfected individuals, prior exposure dose affected mean or heterogeneity in maximum pathogen load or pathology. We used Firth’s logistic regression to test whether primary treatment affected probability of reinfection as there was complete separation (all sham primary treatment became infected during secondary). Using the methods noted above for all challenged birds, we tested for mean differences in maximum pathogen load and eyescore among priming treatments only for the birds successfully infected following secondary challenge. Finally, we calculated PV per treatment group for only the birds that became infected after secondary challenge using each bird’s maximum eyescore and maximum log_10_ pathogen load during secondary challenge. Because only 2/10 birds were successfully reinfected in the high dose priming group, we could not robustly calculate PV for this treatment group and thus we did not include this treatment group in statistical comparisons of variability for successfully infected birds.

Pathogen load is generally log_10_ transformed in this disease system^65^. This scaling emphasizes the relative biological importance of the orders of magnitude and highlights the differences between individuals with low and high pathogen loads. However, transformations change the shape of the data and can affect the quantification of variability by augmenting variation at low values and compacting it at high values. Therefore, while we present our pathogen load data as log_10_-transformed in the main text, we have included variability metrics for both log_10_ transformed and untransformed pathogen load data in the Supplement for comparison (Supplementary Table 5, Supplementary Fig. 2). Importantly, using log_10_ transformed pathogen load did not affect variability metrics for groups with prior exposure. Transforming the data does, however, reduce the CV and PV compared to the raw pathogen load in the control birds that were subsequently inoculated with 7000 CCU/mL MG.

## Supplementary Information

Below is the link to the electronic supplementary material.

Supplementary Material 1.

## Data Availability

Data and code underlying this manuscript are found on the Virginia Tech Data Repository at https://doi.org/10.7294/31852381.
