# Supplementary material for: Prior pathogen exposure augments inter-individual heterogeneity in antibody levels and reinfection loads in a songbird-pathogen system
Source: Sci Rep. 2026 Apr 2;16:15762. doi: 10.1038/s41598-026-46682-9 (PMC13195175; doi:10.1038/s41598-026-46682-9)
Supplement: Supplementary file 1 — Supplementary Material 1. [file 41598_2026_46682_MOESM1_ESM.docx]

## **Supplement**

## **Prior pathogen exposure augments inter-individual heterogeneity in antibody levels and reinfection loads in a songbird-pathogen system**

**Authors:** Jesse N. Garrett-Larsen*^,+,1^, Anna A. Pérez-Umphrey ^+,1^, Arietta E. Fleming-Davies ^2^, James S. Adelman^3^, Lauren M. Childs^4^, Steven J. Geary ^5^, Kate E. Langwig**^++^**^,1^ & Dana M. Hawley^++,1^

^1^ Department of Biological Sciences, Virginia Tech, Blacksburg, Virginia, United States of America, ^2^ Department of Biology, University of San Diego, San Diego, California, United States of America, ^3^ Department of Biological Sciences, University of Memphis, Memphis, Tennessee, United States of America, ^4^ Department of Mathematics and Virginia Tech Center for the Mathematics of Biosystems, Virginia Tech, Blacksburg, Virginia, United States of America, ^5^ Department of Pathobiology & Veterinary Science, University of Connecticut, Storrs, Connecticut, United States of America

*Corresponding Author; jessegl@vt.edu

^+^ These authors contributed equally to this work.

^++^ These authors contributed equally to this work.

## **Supplemental Methods**

### **Field capture & quarantine**

From May to August 2023, 157 wild house finches were caught in Montgomery County, VA using wire cage traps and mist nets at baited feeders. Only hatch year (juvenile) birds without clinical signs of Mycoplasmal conjunctivitis were retained. Birds were pair-housed in indoor cages (46 x 76 x 46 cm) in rooms set to a 12L:12D light cycle and monitored for clinical signs of MG infection (i.e., conjunctivitis) for three weeks upon intake. In the third week of quarantine, blood plasma was collected from the brachial vein to confirm seronegativity for MG IgY antibodies using a commercial ELISA kit (described in methods). To ensure all birds were pathogen-naive, any bird that developed pathology during their quarantine, had an infected cagemate, or was seropositive for MG IgY antibodies, was excluded from the study.

### **Sample Sizes**

Of the 157 wild-caught birds, 7 were excluded from some or all analyses: One died of unknown causes before the completion of the experiment and another was later determined to be seropositive just prior to the start of the experiment, and these were excluded from all analyses. Following primary challenge, birds were considered unrecovered if their eyeswab had a pathogen load greater than 50 copies in a qPCR assay on DPPI [days post-priming inoculation] 41 [1]. Five birds met these criteria directly before reinfection and were excluded from all analyses.

Final sample sizes were n=150 for all analyses of eye score and pathogen load. Fifteen plasma samples were lost from DPPI 14 and one from DPPI 41 sampling, resulting in reduced sample sizes for antibody analyses. Of the fifteen samples lost from DPPI 14, fourteen were sham inoculated and one was inoculated with a low dose. The one plasma sample lost from DPPI 41 was from a bird inoculated with a low dose. Because GLMMs were performed separately for individual sampling days, individuals with missing samples were retained in antibody analyses on days on which they did have samples, resulting in variable sample sizes. Complete removal of individuals with missing plasma samples from all antibody analyses did not meaningfully change effect sizes, significance, means, or variability metrics.

### **Experimental timeline & inoculations**

*Primary exposure*

Three weeks prior to the start of the experiment, birds were moved into clean (46 x 76 x 46 cm) cages and single-housed within the same facility where they were quarantined (Supplemental - Methods). Baseline plasma and eyeswab samples were collected (described below) one week prior to when primary inoculations were administered. To create a gradient of prior exposure, birds were inoculated via ocular absorption with 70 µL (35 µl per eye) of a solution of MG suspended in Frey’s media. Birds received one of three primary treatments: sham control [0.0 CCU/mL; sterile Frey’s media]; low dose [7.5 x 10^2^ CCU/mL]; or high dose [3.0 x 10^4^ CCU/mL] (Fig. 1). The MG strain used was the original index isolate, VA1994 [2]. Beginning one week (DPPI 7) after the inoculations, samples and data were collected weekly until DPPI 41. Measurements of pathology (eyescores), pathogen load (eyeswabs), and antibody data were collected longitudinally for each bird. Because IgY antibody data were collected on DPPI 41 (i.e., one day before secondary inoculations took place), those antibody measurements should relate to a bird’s reinfection susceptibility the following day when they received their secondary exposure dose (below).

*Secondary exposure*

Birds were allowed to recover from their primary exposure for forty-one days, at which point they were checked for unresolved MG infections via eye scoring and swabbing. The next day (DPPI 42), birds from each primary treatment group received one of five secondary challenge doses in the same manner as the primary inoculations: 0.0 CCU/mL (i.e., sham), 3.0 x 10^1^ CCU/mL, 1.0 x 10^2^ CCU/mL, 3.0 x 10^2^ CCU/mL, or 7.0 x 10^3^ CCU/mL (Figure 1). Measurements of pathology (eyescores) and pathogen load data (eyeswabs) were collected beginning four days after secondary challenge (DPSI [days post-secondary inoculation] 4), and then weekly through the end of the study on DPSI 21.

### **Supplemental Tables and Figures**

### **Table S1. Model results for antibody analyses.** Model output for GLMM testing the effect of primary treatment on mean antibody levels on three different days post primary inoculation (DPPI -8, 14, and 41). Estimates for both the conditional model (mean response) and dispersion estimates (variance structure) are presented on the log scale. Reference categories were Primary Treatment = Sham and DPPI = -8. Bolded p-values are significant (ɑ = 0.05).


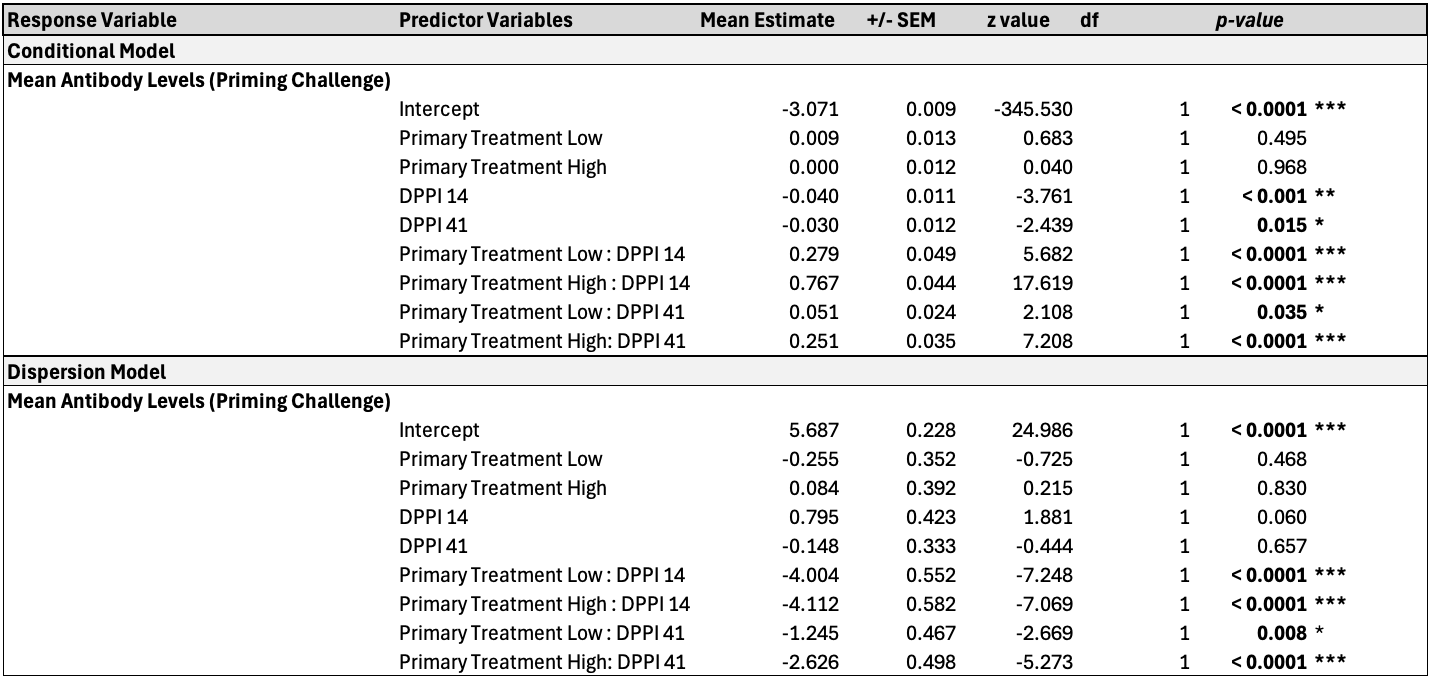


##### **Table S2. Model results for antibody analyses.** Model outputs for two GLMs (susceptibility to reinfection) testing the predictive ability of antibodies for reinfection susceptibility (yes or no) independently on two different days post-priming inoculation (14, and 41). Bolded p-values are significant (ɑ = 0.05).


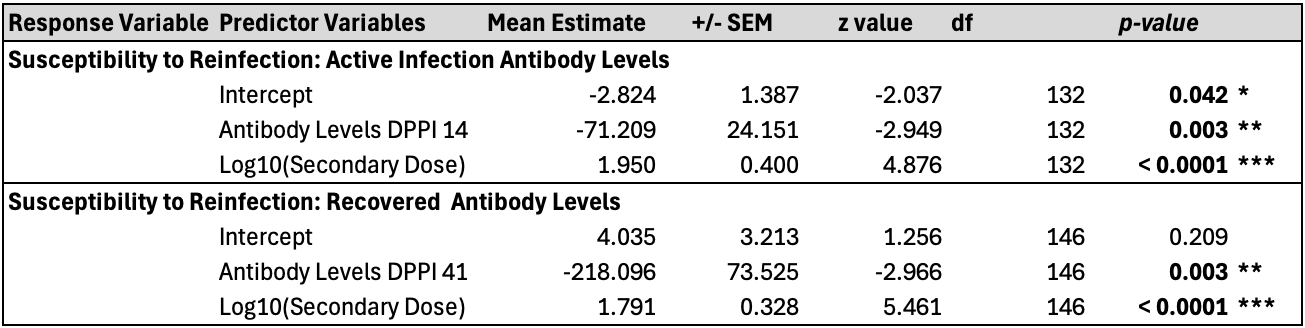


##### **Table S3. Summary of results for eyescore and pathogen load data.** Variability metrics (coefficient of variation [CV] and proportional variability [PV]) calculated for each primary treatment group based on maximum eye score and log_10_ pathogen load following secondary (7,000 dose only) pathogen challenge for all individuals. Mean value per metric (maximum recorded “eyescore” and “pathogen load”), standard error (SE), and number of infected and uninfected individuals are reported. Groups are subset into all birds regardless of reinfection status (top) and reinfected birds only (>50 copies; bottom).

**
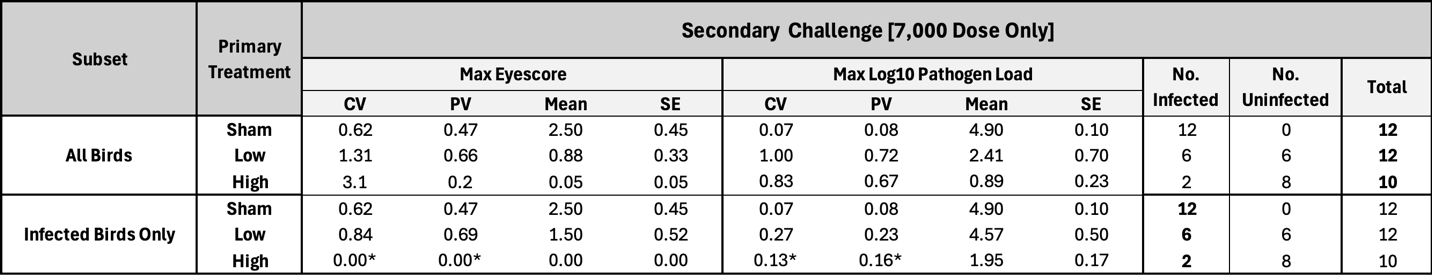
**

**Table S4. Primary and secondary challenge sample sizes.** Number of birds per treatment group. Primary and Secondary Dose columns indicate the pathogen inoculation doses in CCU/mL and the respective number of birds that received each dose. **
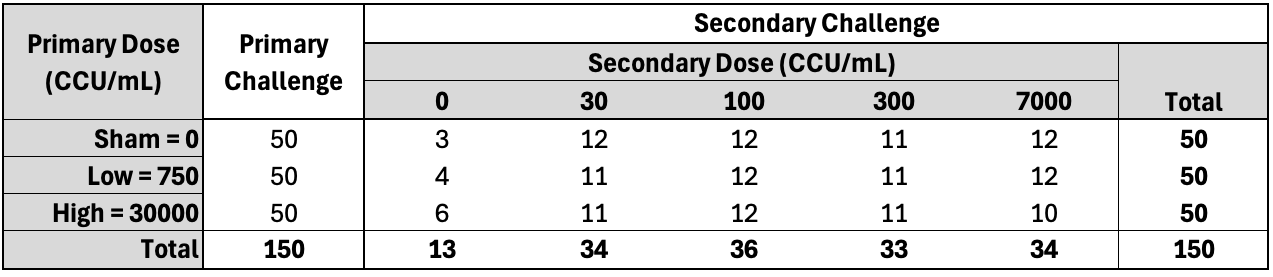
**

**Table S5. Summary of results for raw max pathogen load and log10 transformed max pathogen load data.** Variability metrics (coefficient of variation [CV] and proportional variability [PV]) calculated for each primary treatment group based on maximum raw pathogen load and log_10_ pathogen load following secondary (7,000 dose only) pathogen challenge for all individuals. Mean value per metric (maximum recorded “pathogen load”), standard error (SE), and number of infected and uninfected individuals are reported.

**
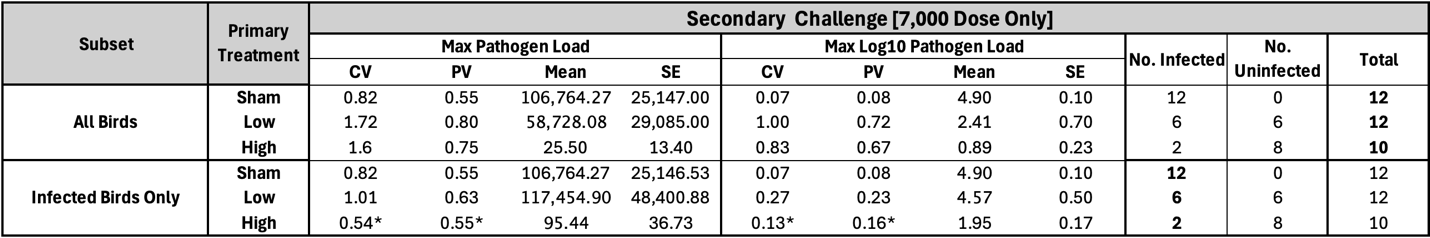
**


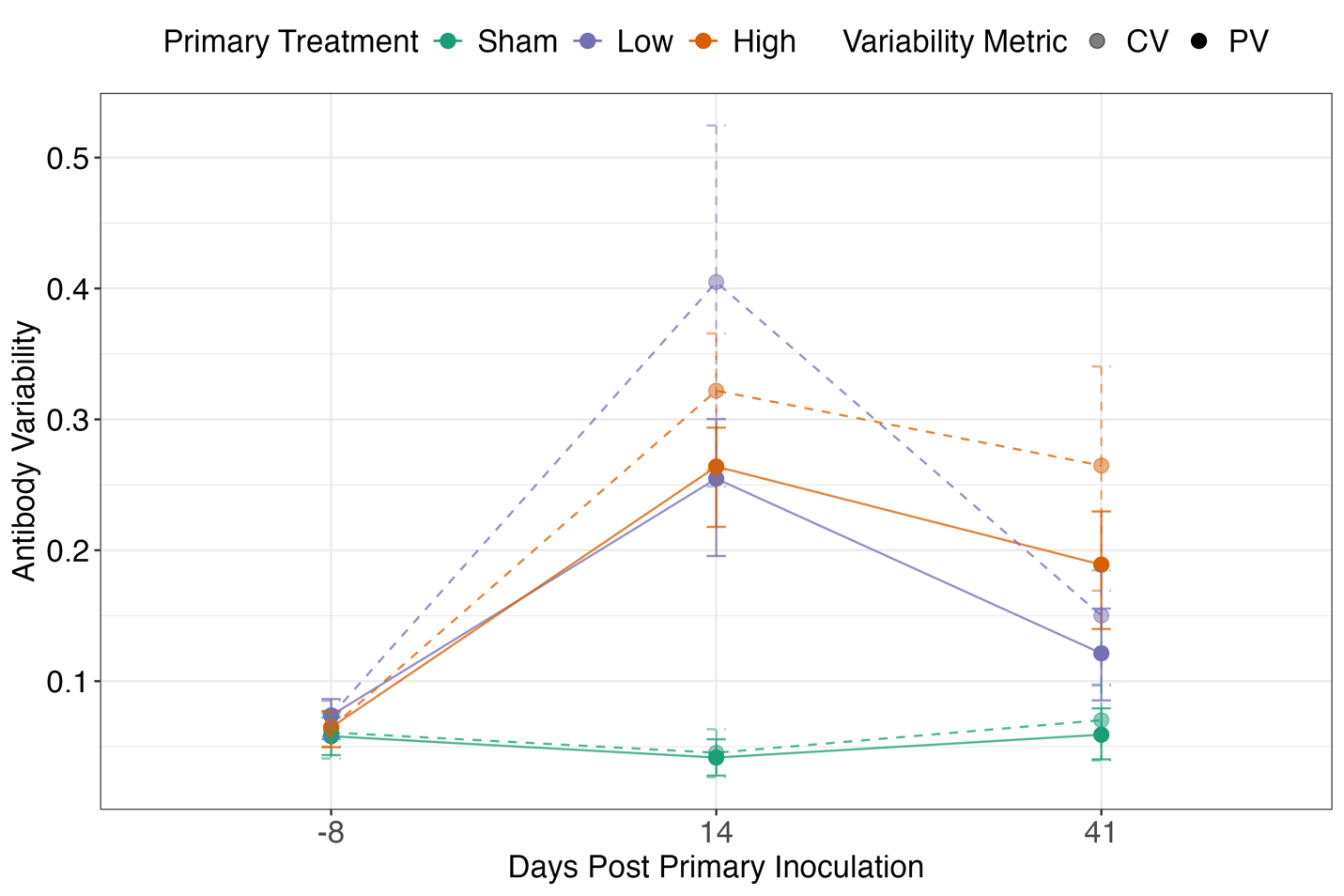


**Figure S1. Comparison of metrics of variability for antibody levels across primary challenge**. Proportional variability (PV; solid points and lines) and coefficient of variation (CV; transparent points and dashed lines) of antibody levels calculated for each primary treatment group per sampling day following primary challenge (days post-priming inoculation 0). Error bars represent 95% CIs calculated by bootstrapping the raw data 1,000 times with replacement.


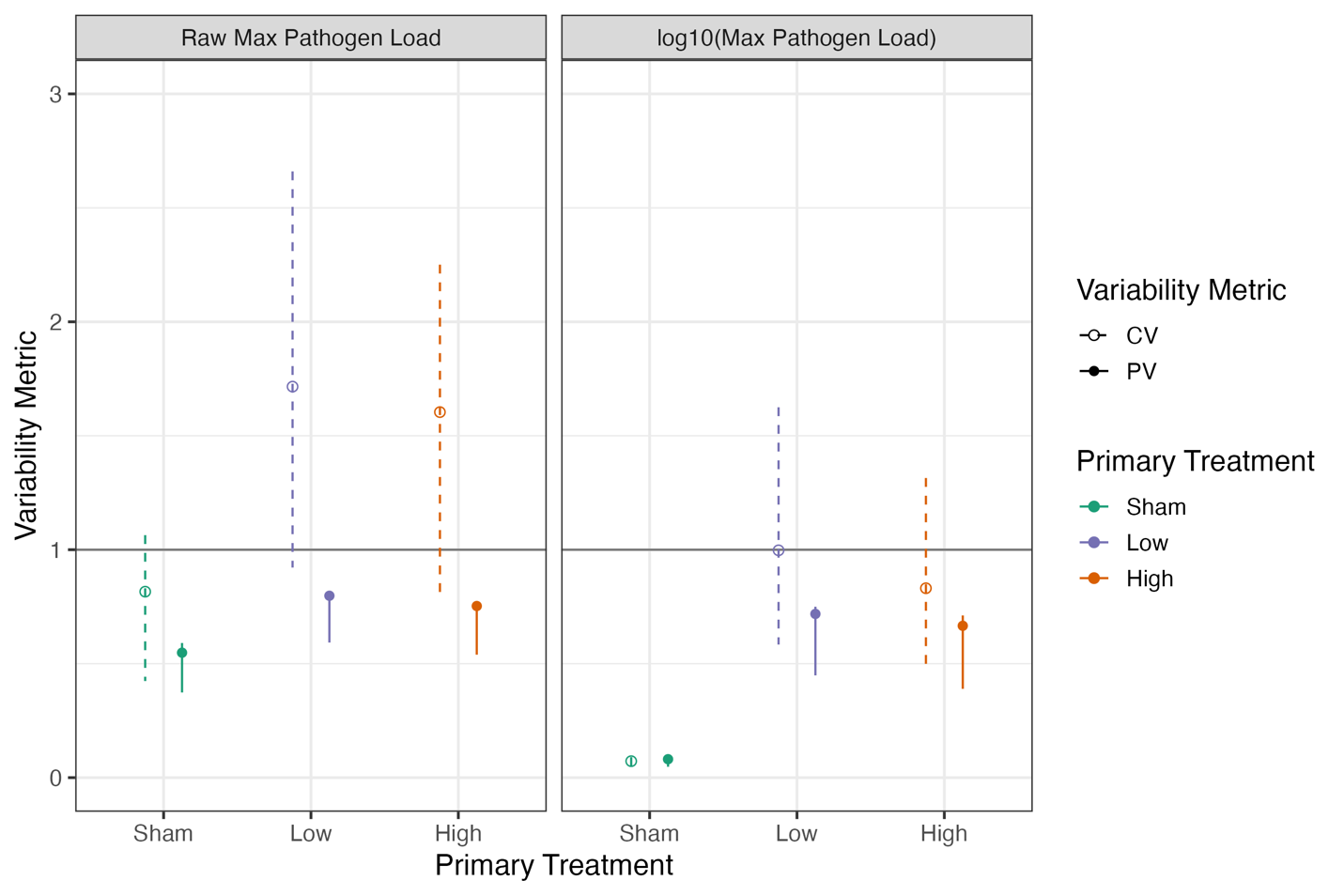


**Figure S2. Comparison of variability metrics between raw and log10 max pathogen load.** Proportional variability (PV; solid points and error bars) and coefficient of variation (CV; hollow points and dashed error bars) of raw and log10 transformed max pathogen loads calculated for each primary treatment group rechallenged with 7,000 CCU/mL MG. Error bars represent 95% CIs calculated by bootstrapping the raw data 1,000 times with replacement.

**References**

1. Hawley, D. M. *et al.* Prior exposure to pathogens augments host heterogeneity in susceptibility and has key epidemiological consequences. *PLOS Pathogens* **20**, e1012092 (2024).

2. Ley, D. H., Berkhoff, J. E. & McLaren, J. M. Mycoplasma gallisepticum Isolated from House Finches (Carpodacus mexicanus) with Conjunctivitis. *Avian Diseases* **40**, 480–483 (1996).
